# Angiotensin-converting enzyme inhibitors during the first trimester of pregnancy increase the incidence of fetal malformation, whereas calcium intake (1.0 to 2.0 g/day) prevents preeclampsia*

**DOI:** 10.1590/S1516-31802006000500001

**Published:** 2006-09-07

A retrospective study on 29,507 pregnant women published in the New England Journal of Medicine in June 2006 showed that the use of angiotensin-converting enzyme (ACE) inhibitors during the first trimester of pregnancy increased the risk of major fetal malformation. The estimated risk ratio was 2.71, with a 95% confidence interval (CI) of 1.72-4.27, in comparison with patients who did not receive anti-hypertensive medication. Other hypertensive drugs utilized during the same period of pregnancy did not increase the risk of malformation.^1^

The fetuses exposed to ACE inhibitors presented around three to four times greater risk of cardiovascular malformation (risk ratio: 3.72; 95% CI: 1.89-7.30) and of central nervous system malformation (risk ratio: 4.39; 95% CI: 1.97-14.02). The authors concluded that exposing pregnant women to ACE inhibitors during the first trimester of pregnancy was unsafe and must be avoided. Other studies had already demonstrated fetal risks in using ACE inhibitors during the second and third trimesters of pregnancy.

The conclusions from the study described here and from previous studies are categorical and it would be difficult for anyone to take an interest in conducting clinical studies on a drug to increase the certainty that it causes fetal malformation.

In order to establish whether a new treatment is better than a previous one (i.e. that it is generally effective), high-quality "megatrials" are needed. But to recommend the exclusion of a drug for which the potential risk is very high and unacceptable, a lower level of evidence is sufficient, which in this case was a retrospective observational study. It is just a question of good sense. And particularly because there are other alternatives for treating arterial hypertension during pregnancy that are safer, such as alpha-methyldopa and hydralazine.

If we consider that we need evidence in order to know what may work and what may **not** work, this question just needs to be answered rationally. For a long time, we had been reading articles saying that there is no evidence that ACE inhibitors cause malformations during the first trimester of pregnancy. Well, now there is. Lack of evidence does not signify absence of effects. Such effects may be good or bad. Hence the need for us to group together reliable scientific data (randomized clinical trials and systematic reviews) before we expose mothers, fetuses, sons, daughters, fathers, siblings, friends and children of God in general, to uncalculated risks. It can be seen that, in this case, because of the lack of evidence from previous clinical trials regarding the use of this drug during pregnancy, many fetuses developed serious malformations. The mistake of not researching the safety of treatments before implementing them has been repeated *ad nauseam*.

## Calcium supplementation during pregnancy

The systematic review on calcium supplementation published in the Cochrane Library has been updated with a new clinical trial. The latest edition of the review presents a total of almost 15,000 cases studied, through the addition of a large clinical trial that was motivated by the publication of an earlier edition of this review. The new trial included was funded by the World Health Organization (WHO) and was conducted by researchers in several countries. The review is now more robust and reliable, and shows that the use of calcium during pregnancy among at-risk mothers with low calcium intake reduces the incidence of preeclampsia by 50%. Moreover, calcium use decreases the incidence of outcomes consisting of death or serious morbidity. No risks to the mother or fetus were demonstrated through calcium use.

## Implications

In summary, an observational study has shown **what must *not* be done:** prescribing of ACE inhibitors during the first trimester of pregnancy (or maintaining such prescriptions after detecting pregnancy). On the other hand, a systematic review enriched by a megatrial has shown **what must be done** to reduce maternal-fetal risks: calcium supplementation. In this way, human health will improve from the outset of uterine life onwards, thanks to good medical science.
